# Supplementary material for: CBFB mutations predict endocrine therapy benefit in estrogen receptor–positive breast cancer
Source: medRxiv. 2026 May 21:2026.05.18.26353467. Preprint. [Version 1] doi: 10.64898/2026.05.18.26353467 (PMC13228653; doi:10.64898/2026.05.18.26353467)
Supplement: Supplement 1 [file media-1.pdf]

Supplementary Material

CBFB mutations predict endocrine therapy benefit in estrogen receptor–positive breast cancer

Yaacov A, Passi G, Gillis R, Katz D, Grinshpun A

Contains 11 supplementary figures and 3 supplementary tables.

Supplementary Figures

Figure S1. Patient flow diagram (CONSORT-style).

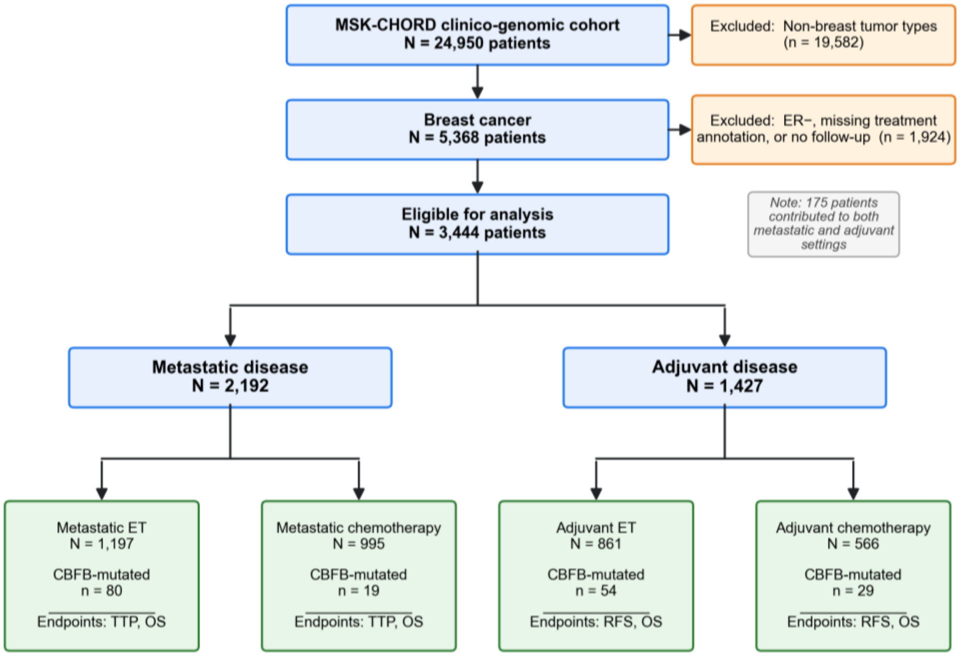

Figure S1. Patient flow diagram (CONSORT-style). Patient flow from the MSK-CHORD source population through cohort definition.

Figure S2. Overall survival by CBFB mutation status in the chemotherapy cohorts (negative controls).

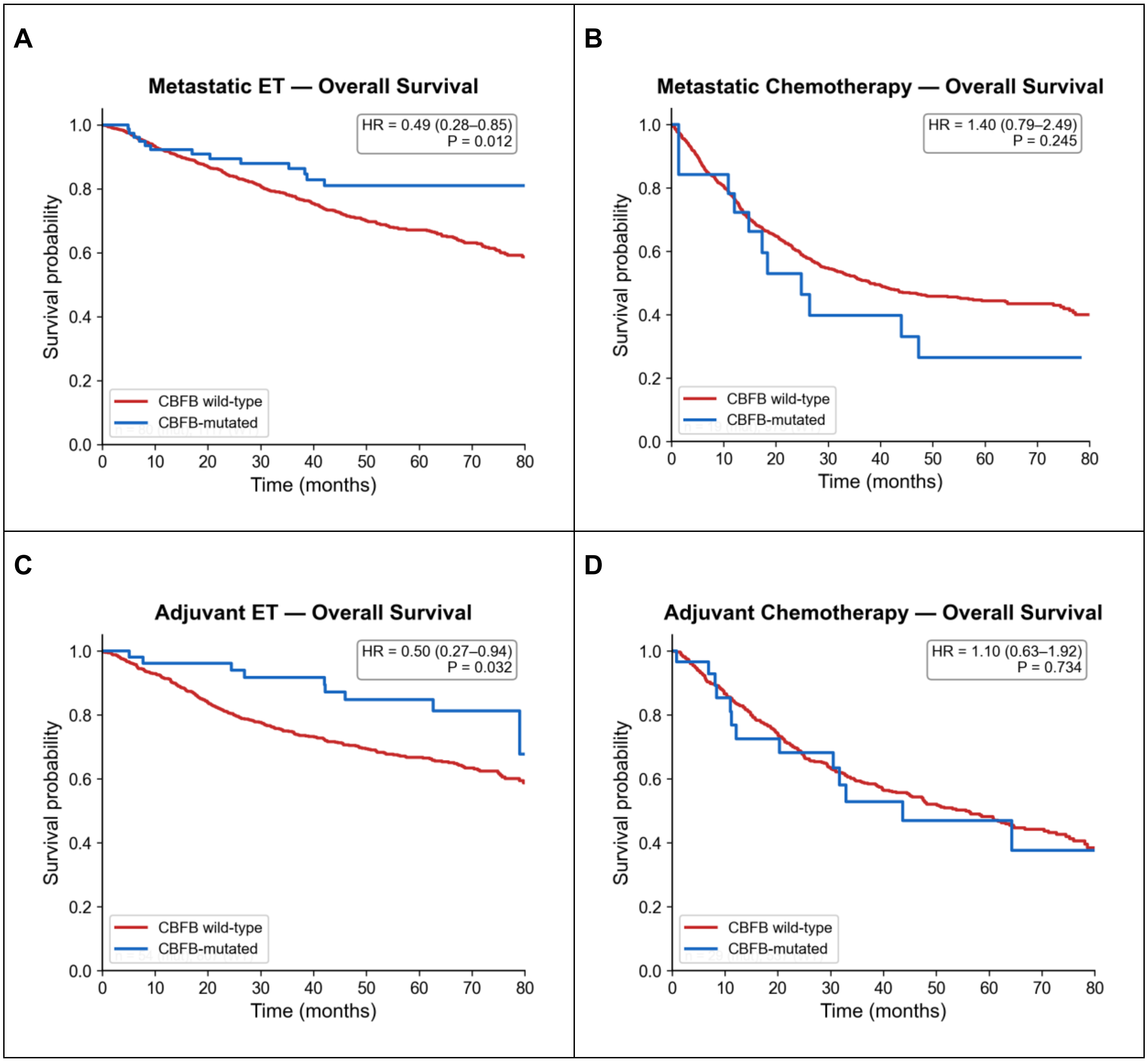

Figure S2. Overall survival by CBFB mutation status in the chemotherapy cohorts (negative controls). Kaplan–Meier curves for overall survival (OS) stratified by CBFB mutation status.

Figure S3. Formal CBFB × PIK3CA interaction test.

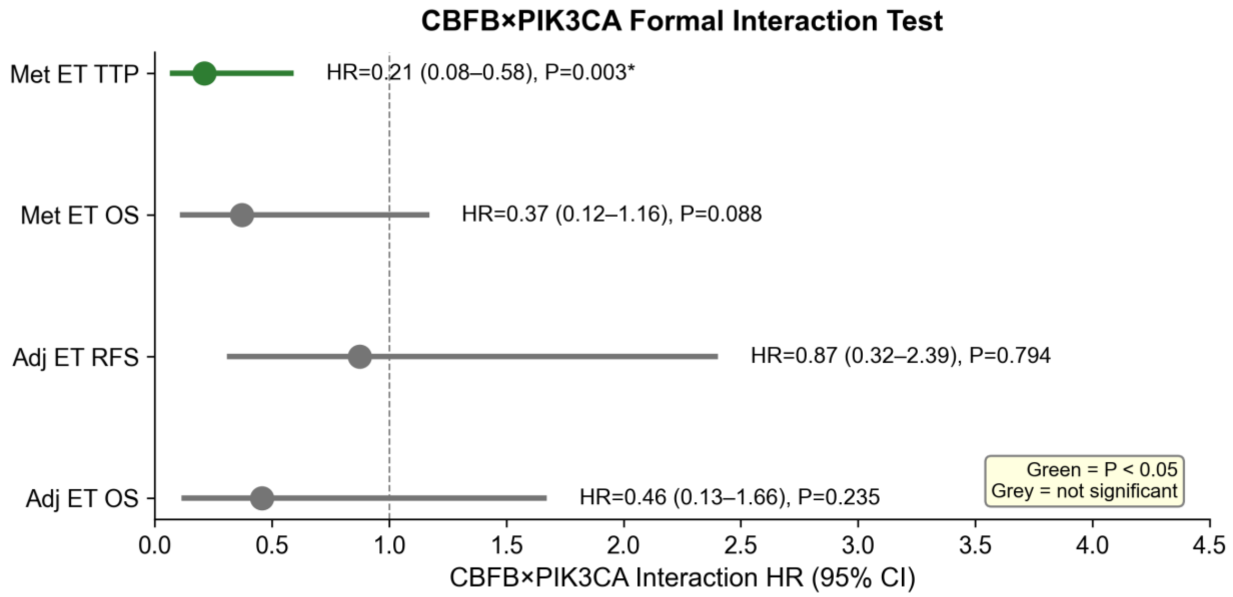

Figure S3. Formal CBFB × PIK3CA interaction test. Forest plot of the formal CBFB × PIK3CA interaction model, adjusted for age and ILC status. The significant interaction (P = .003) supports a cooperative effect of CBFB and PIK3CA loss-of-function on ET sensitivity.

Figure S4. CBF-complex (CBFB + RUNX1) and PIK3CA stratification.

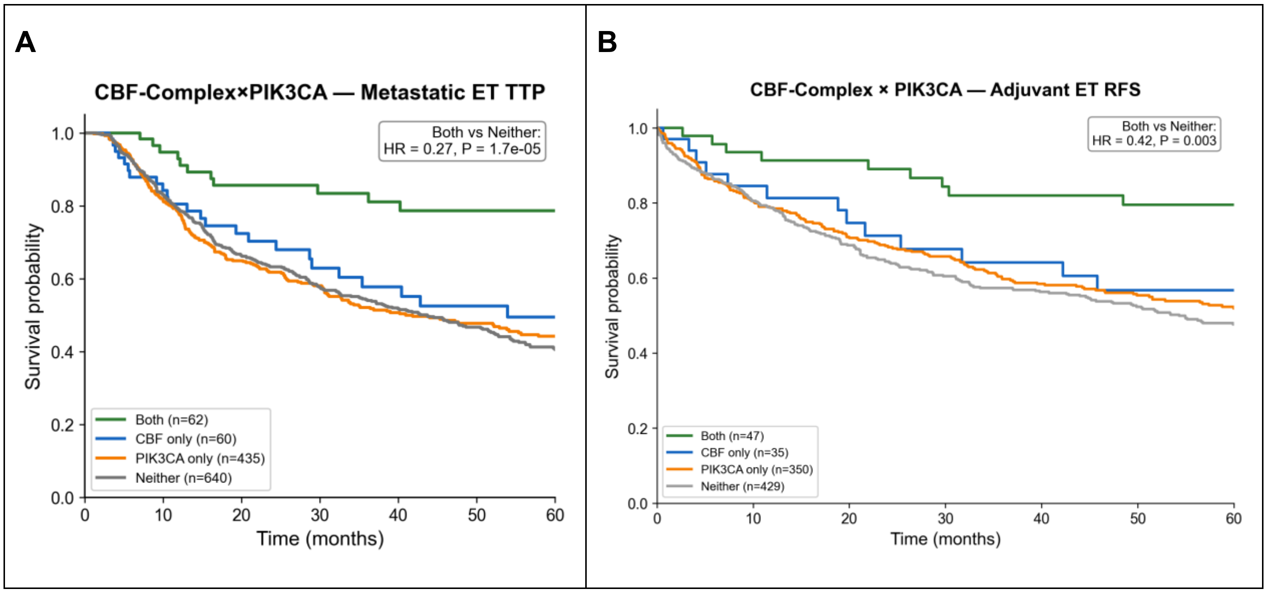

Figure S4. CBF-complex (CBFB + RUNX1) and PIK3CA stratification. Kaplan–Meier curves for the joint CBF-complex × PIK3CA stratification, illustrating that the CBF + PIK3CA co-mutation effect generalizes across the CBF complex. (A) Metastatic ET TTP. (B) Adjuvant ET RFS.

Figure S5. CBFB co-mutation landscape in the metastatic ET cohort.

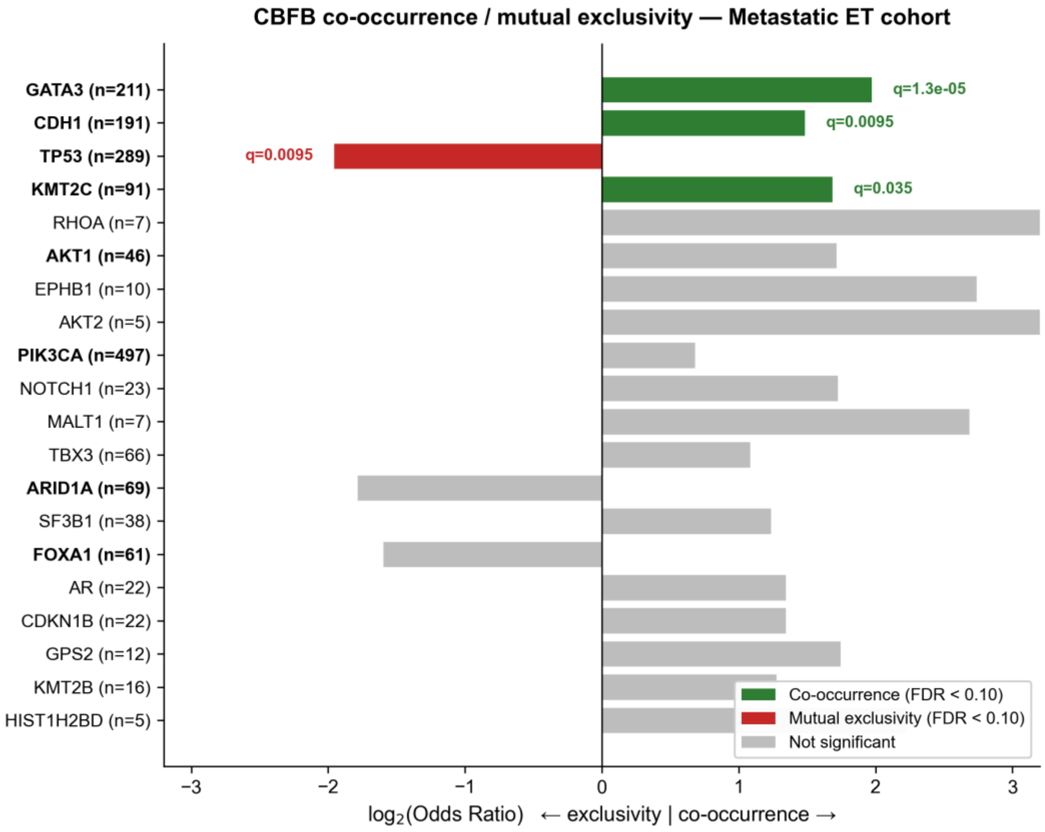

Figure S5. CBFB co-mutation landscape in the metastatic ET cohort. Forest of log2 odds ratios (CBFB-mutated vs CBFB–wild-type) for co-occurrence (positive log2 OR) and mutual exclusivity (negative) across all 240 IMPACT-panel genes meeting the prevalence threshold in the metastatic ET cohort. P values are from two-sided Fisher exact tests; FDR correction by Benjamini–Hochberg. Genes reaching FDR q < .05 are labeled (GATA3, CDH1, KMT2C — co-occurring with CBFB; TP53 — mutually exclusive), consistent with a luminal, ER-axis–intact genomic context for the CBFB-mutated subset.

Figure S6. Left-truncated Cox sensitivity analysis.

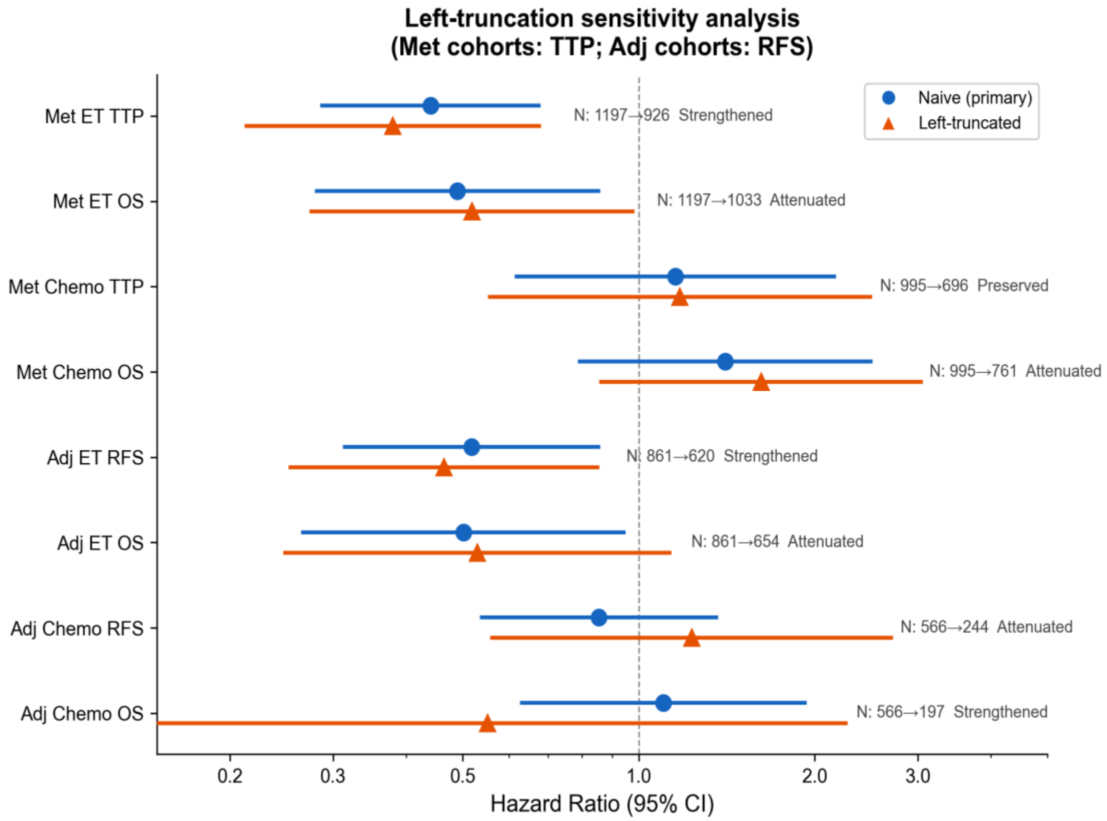

Figure S6. Left-truncated Cox sensitivity analysis. Forest plot comparing naive (left-aligned to first treatment) and left-truncated (delayed-entry at sequencing date) Cox regression hazard ratios across the four cohorts. Hazard ratios for the ET cohorts are preserved or strengthened under left truncation, indicating that the primary findings are not driven by guarantee-time bias.

Figure S7. Subgroup analysis: CBFB effect on TTP in metastatic ET by metastatic pattern.

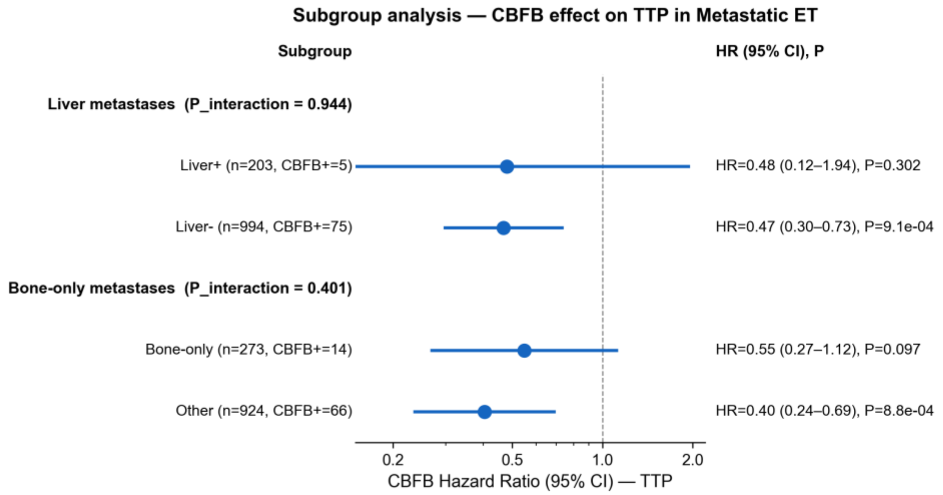

Figure S7. Subgroup analysis: CBFB effect on TTP in metastatic ET by metastatic pattern. Forest plot of CBFB hazard ratios for time to progression in the metastatic ET cohort stratified by (top) liver metastasis status and (bottom) bone-only disease. Subgroup-level HRs with 95% CI and P values are shown; CBFB × subgroup interaction P values test for effect modification. No significant heterogeneity was observed (liver P_interaction = 0.94; bone-only P_interaction = 0.40), supporting that the CBFB benefit generalizes across the dominant metastatic-presentation patterns.

Figure S8. CBFB mutation spectrum.

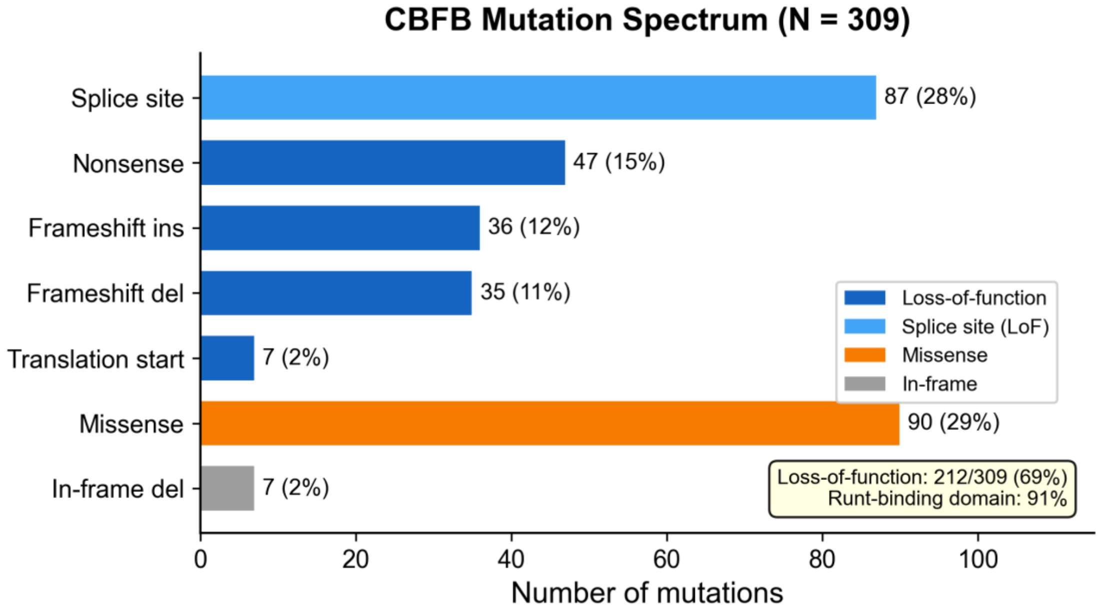

Figure S8. CBFB mutation spectrum. Distribution of CBFB variant classes across the cohort (N = 309 variants in 240 patients). Loss-of-function variants (splice-site, nonsense, frameshift insertion/deletion, translation start site) account for 69% of variants; missense for 29%; in-frame deletions for 2%.

Figure S9. CBFB lollipop plot.

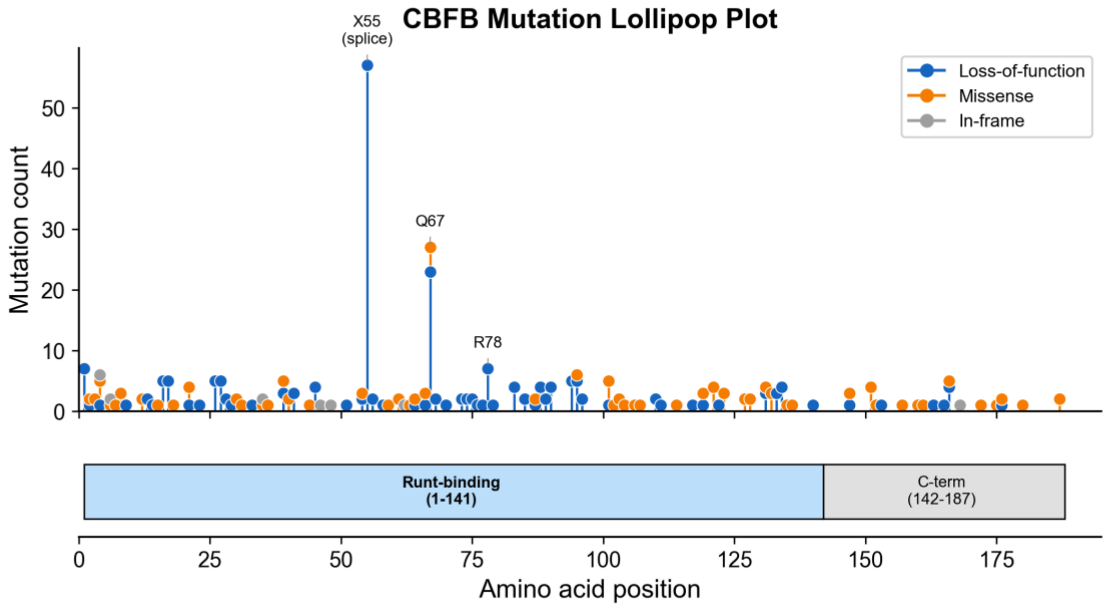

Figure S9. CBFB lollipop plot. Distribution of CBFB variants along the protein, color-coded by variant class. 91% of mutations map to the Runt-binding domain (amino acids 1–141), with a single hotspot at splice-site position 55 accounting for 18% of all mutations.

Figure S10. Mutation-definition sensitivity forest.

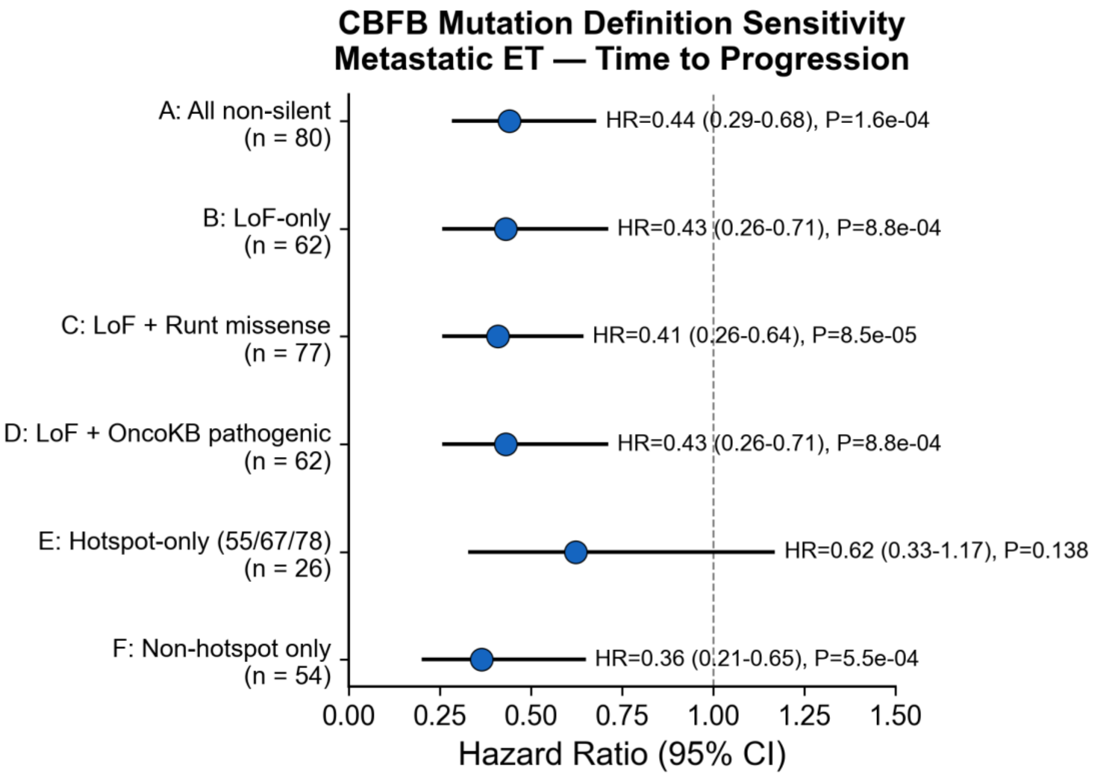

Figure S10. Mutation-definition sensitivity forest. CBFB hazard ratios across alternative mutation definitions: (A) all non-silent (primary); (B) loss-of-function only; (C) LoF + Runt-domain missense; (D) LoF + OncoKB-annotated pathogenic; (E) hotspot-only; (F) non-hotspot only. The narrow HR range (0.41–0.44) across the inclusive definitions confirms that the finding is driven by LoF biology.

Figure S11. Structural mapping of CBFB and RUNX1 variants on the CBFβ–RUNX1 Runt domain–DNA ternary complex.

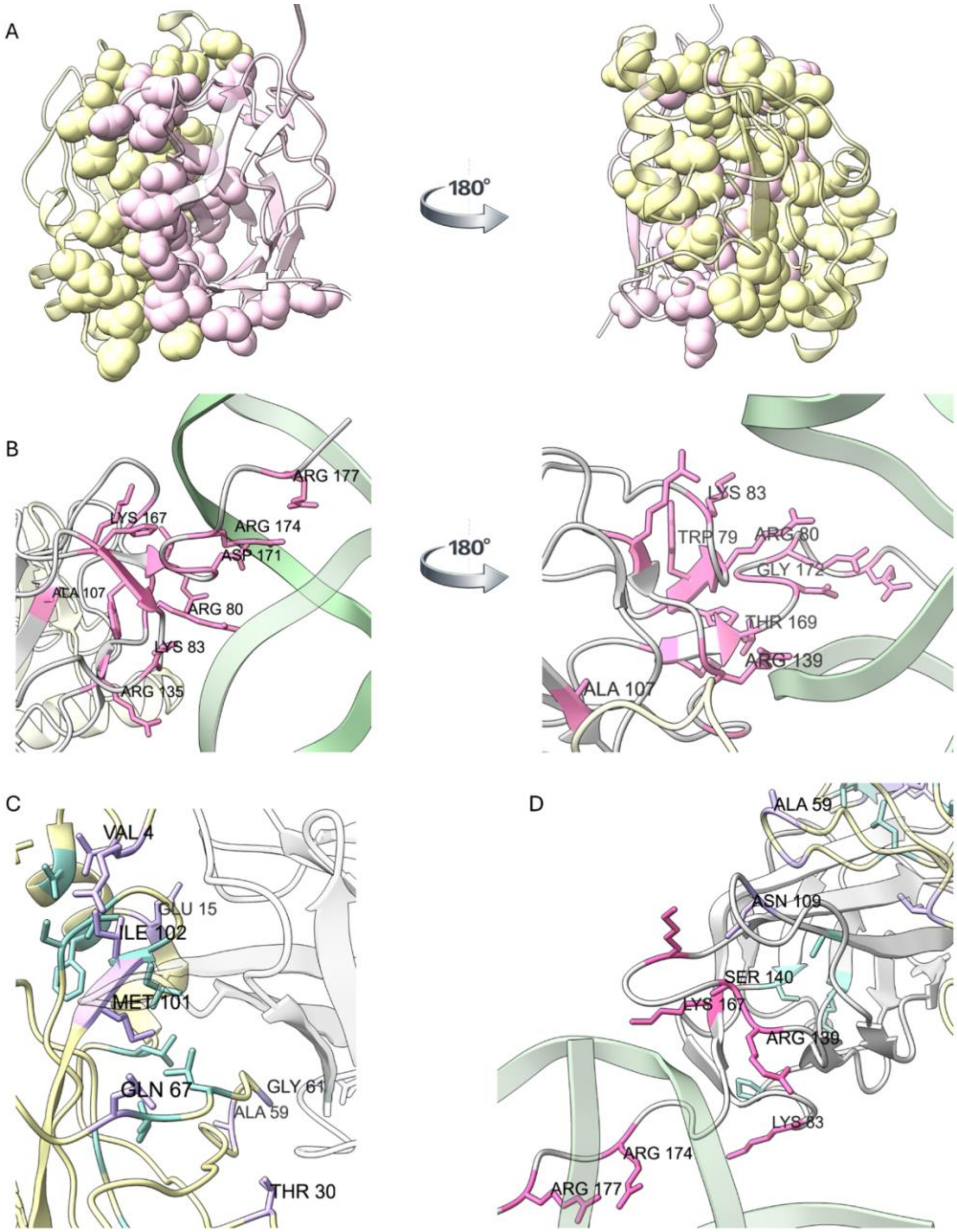

Figure S11. Structural mapping of CBFB and RUNX1 variants on the CBFβ–RUNX1 Runt domain–DNA ternary complex. (A) Interface residues (spheres) in the RUNX1 (yellow) CBFB (pink) complex (PDB 1H9D). (B) Germline pathogenic RUNX1 variants from ClinVar (pink, sticks) mapped onto RUNX1 (gray) DNA (green) and CBFB (yellow) interfaces (PDB 1IO4). (C-D) Somatic CBFB missense variants from MSK-CHORD mapped onto the CBFB-RUNX1 interfaces (purple, sticks), and RUNX1-DNA interface (pink, sticks), other variants shown in blue (PDB 1H9D).

Supplementary Tables

Table ST1. Baseline characteristics by CBFB mutation status across the four cohorts.

Cohort	Variable	CBFB mut	WT	P
Met ET	Age (median)	58	60	0.89
Met ET	N_met_sites (median)	1	2	$<10^{-15}$
Met ET	Liver mets (%)	6.2%	17.7%	0.01
Met ET	Bone mets (%)	25.0%	45.5%	6.0×10^{-4}
Met ET	ILC (%)	25.0%	12.4%	0.003
Met ET	TP53 (%)	7.5%	25.3%	5.0×10^{-4}
Met ET	PIK3CA (%)	52.5%	40.7%	0.05
Adj ET	Age (median)	69	65	0.14
Adj ET	ILC (%)	7.4%	11.3%	0.51
Adj ET	TP53 (%)	9.3%	21.9%	0.04
Adj ET	PIK3CA (%)	59.3%	45.2%	0.06

P values from Wilcoxon rank-sum (continuous) or Fisher exact (categorical) tests. No clinically meaningful imbalances were observed between CBFB-mutated and CBFB-wild-type patients.

Table ST2. Multivariable Cox models for CBFB across treatment cohorts.

Model	Variable	N	HR	CI lower	CI upper	P
Met ET TTP — Age-adjusted	CBFB	1197	0.44	0.29	0.67	1.6×10^{-4}
Met ET TTP — Age-adjusted	CURRENT_AGE_DEID	1197	1.00	0.99	1.01	0.89
Met ET OS — Age-adjusted	CBFB	1197	0.49	0.28	0.86	0.01
Met ET OS — Age-adjusted	CURRENT_AGE_DEID	1197	1.01	1.00	1.02	0.07
Adj ET RFS — Age-adjusted	CBFB	861	0.53	0.32	0.88	0.01
Adj ET RFS — Age-adjusted	CURRENT_AGE_DEID	861	0.98	0.98	0.99	6.9×10^{-6}
Adj ET OS — Age-adjusted	CBFB	861	0.52	0.28	0.97	0.04
Adj ET OS — Age-adjusted	CURRENT_AGE_DEID	861	0.98	0.97	0.99	9.8×10^{-5}
Met ET TTP — Age+ILC-adjusted	CBFB	1197	0.46	0.30	0.70	3.7×10^{-4}
Met ET TTP — Age+ILC-adjusted	CURRENT_AGE_DEID	1197	1.00	0.99	1.01	0.73
Met ET TTP — Age+ILC-adjusted	IS_ILC	1197	0.70	0.54	0.91	0.008
Met ET OS — Age+ILC-adjusted	CBFB	1197	0.49	0.28	0.85	0.01
Met ET OS — Age+ILC-adjusted	CURRENT_AGE_DEID	1197	1.01	1.00	1.01	0.08
Met ET OS — Age+ILC-adjusted	IS_ILC	1197	1.05	0.79	1.41	0.72
Adj ET RFS — Age+ILC-adjusted	CBFB	861	0.54	0.32	0.88	0.01
Adj ET RFS — Age+ILC-adjusted	CURRENT_AGE_DEID	861	0.98	0.98	0.99	5.3×10^{-6}
Adj ET RFS — Age+ILC-adjusted	IS_ILC	861	1.12	0.83	1.51	0.45

Model	Variable	N	HR	CI lower	CI upper	P
Adj ET OS — Age+ILC-adjusted	CBFB	861	0.52	0.28	0.98	0.04
Adj ET OS — Age+ILC-adjusted	CURRENT_AGE_DEID	861	0.98	0.97	0.99	9.5×10^{-5}
Adj ET OS — Age+ILC-adjusted	IS_ILC	861	1.07	0.73	1.55	0.74
Met ET TTP — Full genomic	CBFB	1197	0.50	0.33	0.77	0.002
Met ET TTP — Full genomic	CURRENT_AGE_DEID	1197	1.00	1.00	1.01	0.57
Met ET TTP — Full genomic	IS_ILC	1197	0.78	0.60	1.01	0.06
Met ET TTP — Full genomic	TP53	1197	1.69	1.41	2.02	2.1×10^{-8}
Met ET TTP — Full genomic	ESR1	1197	2.03	1.53	2.70	1.2×10^{-6}
Met ET OS — Full genomic	CBFB	1197	0.53	0.31	0.93	0.03
Met ET OS — Full genomic	CURRENT_AGE_DEID	1197	1.01	1.00	1.02	0.04
Met ET OS — Full genomic	IS_ILC	1197	1.28	0.95	1.72	0.11
Met ET OS — Full genomic	TP53	1197	2.21	1.76	2.79	1.7×10^{-11}
Met ET OS — Full genomic	ESR1	1197	3.59	2.59	4.98	1.8×10^{-14}

Nested multivariable Cox proportional hazards models. Model A: CBFB + age. Model B: + ILC histology. Model C: + TP53 + ESR1. HR = hazard ratio for CBFB mutation; 95% CI = 95% confidence interval.

Table ST3. CBF-complex (CBFB or RUNX1) biomarker and CBF × PIK3CA co-mutation results.

Cohort	Endpoint	Analysis	Group	N	n group	HR	CI lower	CI upper	P
Met ET	TTP	CBF univariate	CBF_COMPLEX	1197	122	0.48	0.34	0.67	2.0×10^{-5}
Met ET	TTP	CBF×PIK3CA 4-group	CBF_only	1197	60	0.73	0.49	1.10	0.13
Met ET	TTP	CBF×PIK3CA 4-group	PIK3CA_only	1197	435	0.99	0.84	1.18	0.95
Met ET	TTP	CBF×PIK3CA 4-group	Both	1197	62	0.27	0.15	0.49	1.7×10^{-5}
Met ET	OS	CBF univariate	CBF_COMPLEX	1197	122	0.60	0.40	0.91	0.02
Met ET	OS	CBF×PIK3CA 4-group	CBF_only	1197	60	0.78	0.45	1.34	0.37
Met ET	OS	CBF×PIK3CA 4-group	PIK3CA_only	1197	435	1.27	1.02	1.58	0.03
Met ET	OS	CBF×PIK3CA 4-group	Both	1197	62	0.57	0.31	1.04	0.07
Met Chemo	TTP	CBF univariate	CBF_COMPLEX	995	45	0.94	0.63	1.40	0.76
Adj ET	RFS	CBF univariate	CBF_COMPLEX	861	82	0.60	0.41	0.88	0.009
Adj ET	RFS	CBF×PIK3CA 4-group	CBF_only	861	35	0.88	0.53	1.48	0.634
Adj ET	RFS	CBF×PIK3CA 4-group	PIK3CA_only	861	350	0.96	0.79	1.15	0.642
Adj ET	RFS	CBF×PIK3CA 4-group	Both	861	47	0.45	0.26	0.78	0.005
Adj ET	OS	CBF univariate	CBF_COMPLEX	861	82	0.76	0.49	1.19	0.23

Cohort	Endpoint	Analysis	Group	N	n group	HR	CI lower	CI upper	P
Adj ET	OS	CBF×PIK3CA 4-group	CBF_only	861	35	1.33	0.77	2.30	0.31
Adj ET	OS	CBF×PIK3CA 4-group	PIK3CA_only	861	350	0.92	0.71	1.18	0.50
Adj ET	OS	CBF×PIK3CA 4-group	Both	861	47	0.39	0.18	0.82	0.01
Adj Chemo	RFS	CBF univariate	CBF_COMPLEX	566	51	0.83	0.58	1.19	0.302

CBF-complex biomarker = patients with mutations in CBFB or RUNX1. Results presented for univariate Cox regression and four-way CBF × PIK3CA stratification.
